# The Malaria Problem

**Published:** 1899-08-12

**Authors:** 


### THE MALARIA PROBLEM.

In his address at Portsmouth last week Dr. Thin gave an account of the present position of the malaria problem and the mosquito theory. The feature of the year in regard to these questions had been the discovery of the exact means by which man becomes infected. The life history of the parasite within the blood of man and within the body of the mosquito had already been traced, and it was obvious that the mosquito became infected by sucking malarious blood. But how the parasite was carried back again to a fresh host, *i.e.*, how man became infected, remained a question. Dr. Manson had thought it probable that the water in which the infected mosquitos died became contaminated with the spores of the malaria parasite, which thus might be taken into the human body, or they might be breathed in the dust of dried up pools. During the past year, however, it had been proved by actual experiment that infected mosquitos—and all kinds of mosquitos do not seem to be capable of carrying the disease—when allowed to feed on susceptible persons are capable of conveying the infection to them. Thus we now have plainly displayed the complete lifecycle of the malarial parasite together with the mechanism of its transit from man to mosquito and from mosquito to man. What we now want to know is the particular sort of mosquito by which each form of fever is carried, and this is what Major Ross has gone out to Africa to discover. We may add, however, that even then we shall not know the complete history of the parasite as it exists in nature, for we can hardly look upon man as its natural host. It cannot be doubted that the natural cycle of the life of the organism lies between the mosquito and the creature whose blood the mosquito naturally sucks, and this is certainly not man. On his first entry into virgin forests, where human feet have never before trodden, man may be attacked by the disease complete in every detail, and we may be quite sure that when the disease is thus endemic man has taken no part in its development. When he is attacked he does but take the place of some other creature who had before served as the host of the parasite alternately with the mosquito, and thus the life history of the organism will not be complete until we know what is this other creature in whose blood the parasite normally has its being. When we know this, perhaps we shall be able to understand more clearly than we do at present why it is that only in certain places do mosquitos prove infective agents. It is not, then, in regard to the mosquito alone that we want information, but as to the creature on which it naturally feeds.

